# Staphylococcus aureus Infective Endocarditis Complicated by Embolic Stroke and Discitis in a Hypertensive Patient

**DOI:** 10.7759/cureus.84215

**Published:** 2025-05-16

**Authors:** Loay Khair

**Affiliations:** 1 Cardiology, Security Forces Hospital, Makkah, SAU

**Keywords:** aortic valve vegetation, discitis, embolization, infective endocarditis, staphylococcus aureus, stroke

## Abstract

Infective endocarditis (IE) remains a severe and potentially fatal condition characterized by high morbidity and mortality. We report a case of a 65-year-old hypertensive male patient who presented with fever, nausea, vomiting, and epigastric pain. Subsequently, he developed an altered mental status. Imaging revealed multiple embolic infarctions. Further investigations confirmed methicillin-sensitive *Staphylococcus aureus* (MSSA) bacteremia with large aortic valve vegetation and a periaortic abscess. Additionally, lumbar spine MRI showed signs suggestive of discitis with neural compression. This case highlights the importance of early recognition of systemic embolization and spinal involvement in IE.

## Introduction

Infective endocarditis (IE) is a life-threatening disease associated with substantial morbidity and mortality despite advances in medical therapy. Its incidence ranges from three to 10 cases per 100,000 people per year, with mortality rates as high as 25%-30% in some studies. Infective endocarditis typically begins with endothelial damage to the heart valves or endocardium. This damage promotes platelet and fribrin deposition, forming a nonbacterial thrombotic endocardial lesion. When bacteremia occurs, circulating microbes adhere to this site, leading to colonization and the formation of infective vegetations. *Staphylococcus aureus* has emerged as the predominant causative organism, particularly among older adults and patients with healthcare-associated infections [1,2].

Systemic embolization occurs in up to 50% of IE cases and may involve the central nervous system, spleen, kidneys, or musculoskeletal system. Stroke, particularly embolic in nature, represents a devastating complication, often associated with large vegetations and increased mortality [1-3]. Vertebral osteomyelitis and discitis due to septic embolization, although less common, have been increasingly recognized and can cause significant morbidity if diagnosis is delayed [4].

Early detection of complications through imaging modalities such as transesophageal echocardiography (TEE) and MRI, alongside timely antimicrobial therapy and surgical consultation, are critical to optimize patient outcomes [1,2]. Herein, we present a rare and complex case of *S. aureus* infective endocarditis complicated by embolic stroke and lumbar discitis, emphasizing the importance of a multidisciplinary approach.

## Case presentation

A 65-year-old male patient with a known history of hypertension presented to Security Forces Hospital Makkah with a three-day history of nausea, vomiting, fever, back pain, and epigastric pain. On admission, the patient appeared fatigued, jaundiced, and required assistance in a wheelchair. His vital signs revealed a blood pressure of 106/63 mmHg, a heart rate of 94 bpm, a respiratory rate of 22 breaths per minute, and a temperature initially 36.8 degrees Celsius, later peaking at 37.8 degrees Celsius.

Physical examination showed an unremarkable cardiovascular assessment (normal S1 and S2, no audible murmurs), a clear chest, a soft and non-tender abdomen without organomegaly, and a neurologically intact initial examination. Laboratory investigations revealed: leukocytosis (white blood count (WBC), 15.89 ×10^9^/L, neutrophils, 88.35%), hemoglobin, 15.0 g/dL, thrombocytopenia (platelet count, 41 ×10^9^/L), serum creatinine at 278.8 µmol/L, and elevated C-reactive protein (CRP, 289.33 mg/L). Blood cultures subsequently grew methicillin-sensitive *S. aureus* (MSSA) (Table 1).

**Table 1:** Laboratory Findings MSSA: methicillin-sensitive *Staphylococcus aureus.*

Laboratory Parameter	Patient Value	Normal Range
White Blood Cell	15.89 ×10⁹/L	4.0–11.0 ×10⁹/L
Neutrophil Percentage	88.35%	40–75%
Hemoglobin	15.0 g/dL	13.5–17.5 g/dL (males)
Platelet Count	41 ×10⁹/L	150–400 ×10⁹/L
Serum Creatinine	278.8 µmol/L	62–115 µmol/L (males)
Blood Culture	MSSA (positive)	No growth / negative

**Figure 1:**
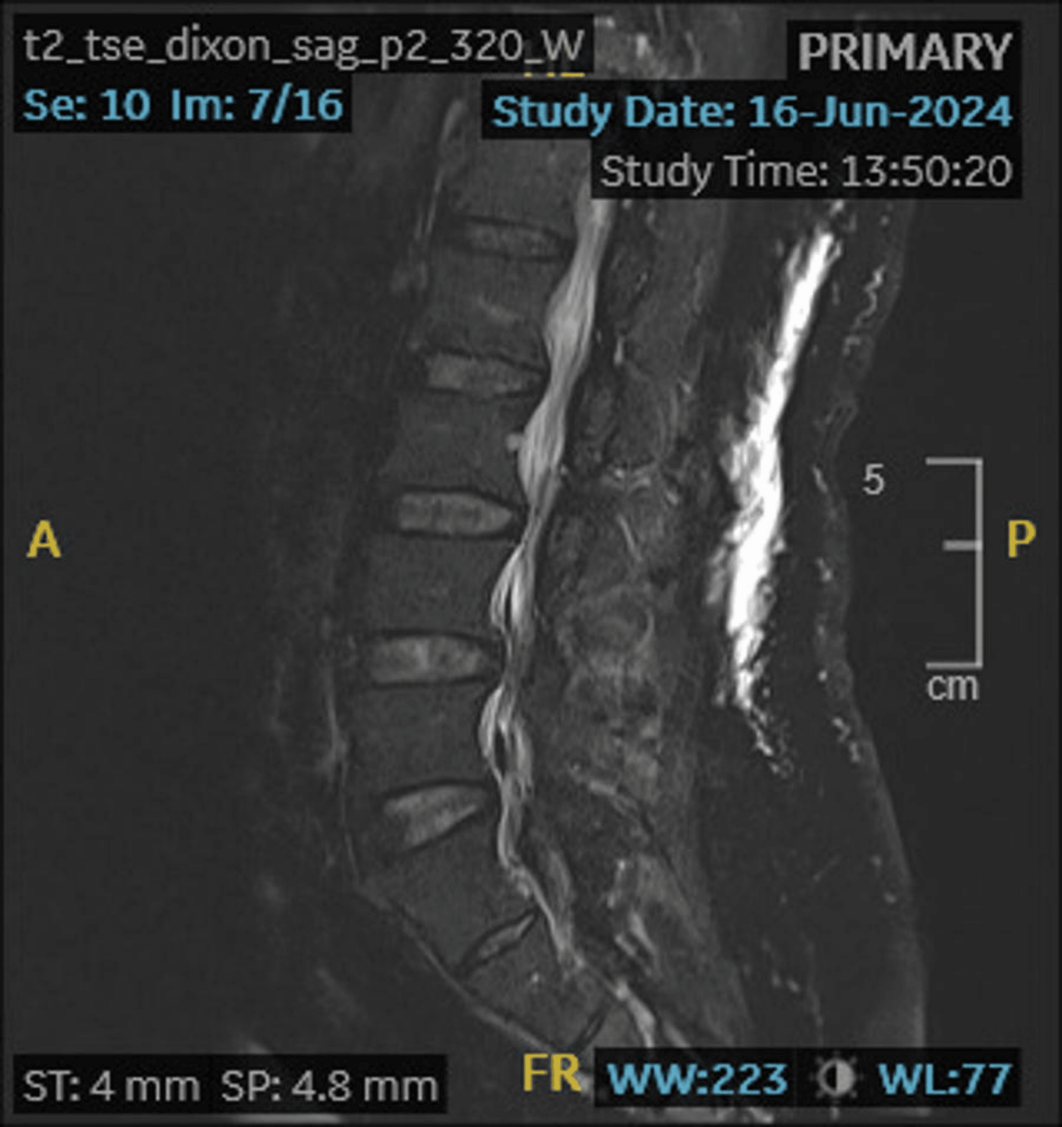
MRI Spine showing disc lesions L3/L4, L4/5 with neural compression

During hospitalization, the patient complained of worsening lower back pain radiating to the buttocks, without lower limb weakness and fecal or urinary incontinence. Lumbar spine MRI showed disc lesions at L3/4 and L4/5 levels with evidence of neural compression, suggestive of discitis (Figure 1).

**Figure 2:**
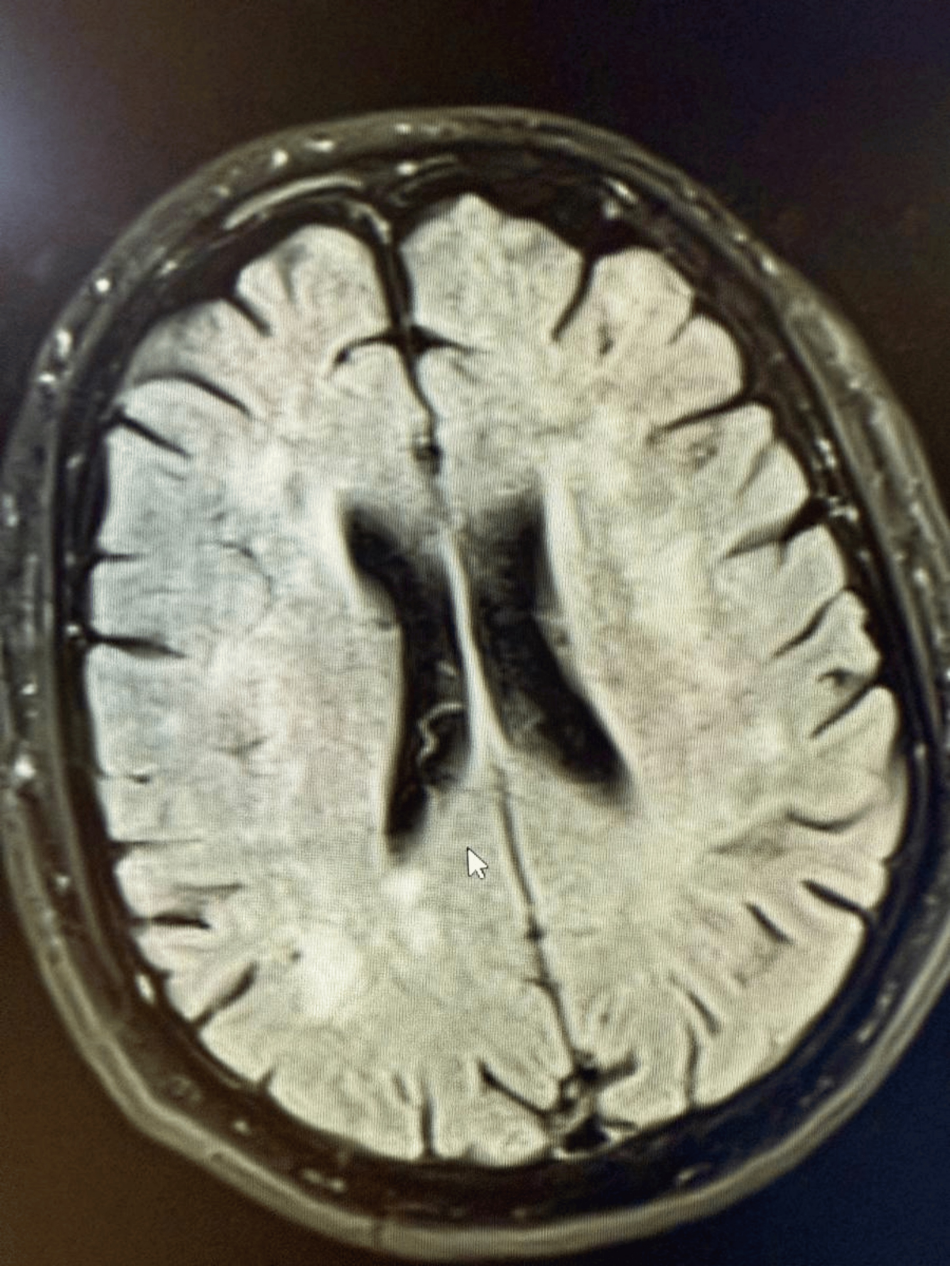
MRI of the brain showing acute infarctions

One day post-admission, the patient developed disorientation and lethargy. Brain CT and MRI revealed multiple embolic infarctions affecting the bilateral centrum semiovale, temporal lobes, cerebellum, and right frontal cortex (Figure 2).

**Figure 3:**
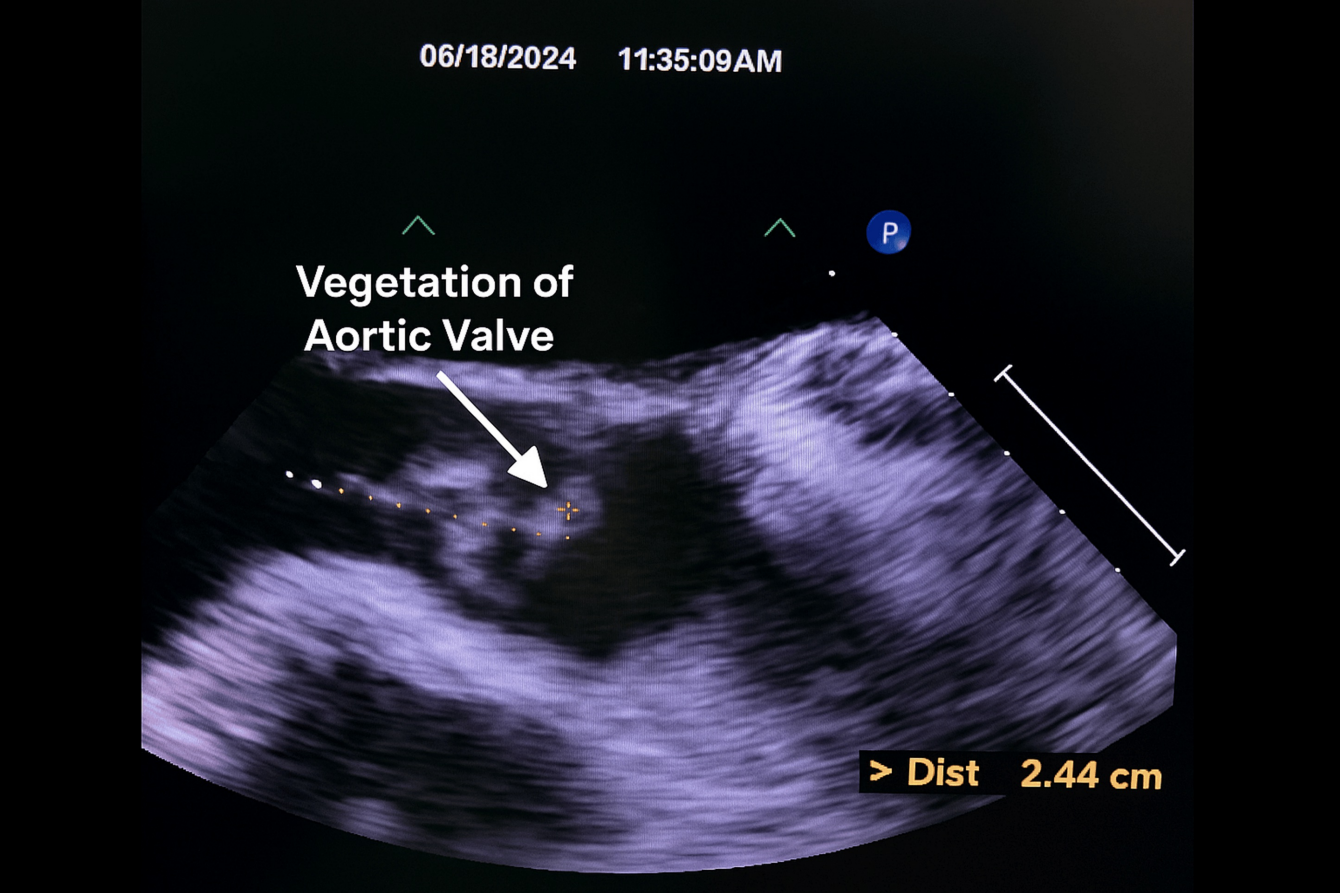
Right coronary cusp (RCC) aortic valve vegetation (transesophageal echocardiography)

Renal ultrasonography was unremarkable. A transthoracic echocardiogram showed mild aortic regurgitation. However, transesophageal echocardiography (TEE) revealed a large (2.4 cm) mobile vegetation attached to the aortic valve (Figure 3), along with a periaortic abscess and moderate aortic regurgitation.

The hospital management included the following: The patient was admitted for five days. During these days, he received the following treatment: administration of 12 units of platelet transfusion; initiation of empirical antibiotics (ceftriaxone and teicoplanin); and adjustment to intravenous cloxacillin after confirmation of MSSA bacteremia. The patient was referred to King Abdullah Medical City (KAMC) for multidisciplinary cardiac surgery evaluation and management in a hemodynamically stable condition. At KAMC, aortic valve replacement (AVR) by bioprosthetic valve was done successfully and the patient was later discharged in a stable condition.

## Discussion

Infective endocarditis due to *S. aureus* is aggressive and often complicated by embolic events and metastatic infections. Embolic strokes occur most frequently during the first week of antimicrobial therapy and are often associated with large vegetations (>10 mm) [1,2].

Discitis and vertebral osteomyelitis are rare but increasingly reported complications of IE. Hematogenous spread through septic emboli can lead to infection of the intervertebral discs and adjacent vertebral bodies [4]. Clinical suspicion should be raised in patients with back pain, even in the absence of classic neurological signs.

Transesophageal echocardiography remains the gold standard for detecting cardiac vegetations, abscesses, and prosthetic valve involvement [1,2]. In our patient, the TEE findings of large vegetation and periaortic abscess necessitated urgent referral for surgical evaluation, considering the high risk of further embolization and hemodynamic deterioration.

Antimicrobial therapy targeting the causative organism remains the cornerstone of IE treatment. Cloxacillin is preferred for MSSA endocarditis due to its superior efficacy compared to vancomycin [1,2]. Surgical intervention should be considered in patients with heart failure, persistent bacteremia, large vegetations, or periannular complications [1,2].

## Conclusions

This case underscores the aggressive nature of *Staphylococcus aureus* infective endocarditis and the potential for devastating multi-systemic complications such as embolic stroke and discitis. Early imaging, prompt initiation of appropriate antimicrobial therapy, and timely surgical consultation are crucial for improving patient outcomes. Clinicians should maintain a high index of suspicion for embolic and metastatic complications in patients presenting with neurological symptoms or persistent back pain.
